# Tracking the Emergence of a New Breed Using 49,034 SNP in Sheep

**DOI:** 10.1371/journal.pone.0041508

**Published:** 2012-07-27

**Authors:** James W. Kijas, James E. Miller, Tracy Hadfield, Russell McCulloch, Elsa Garcia-Gamez, Laercio R. Porto Neto, Noelle Cockett

**Affiliations:** 1 Division of Livestock Industries, Commonwealth Scientific and Industrial Research Organisation, Brisbane, Queensland, Australia; 2 Department of Pathobiological Sciences, School of Veterinary Medicine and Department of Veterinary Science, Louisiana State University, Baton Rouge, Louisiana, United States of America; 3 Department of Animal, Dairy, and Veterinary Sciences, Utah State University, Logan, Utah, United States of America; 4 Departmento de Produccion Animal, Universidad de Leon, Leon, Spain; 5 School of Veterinary Science, The University of Queensland, Gatton, Queensland, Australia; BiK-F Biodiversity and Climate Research Center, Germany

## Abstract

Domestic animals are unique in that they have been organised into managed populations called breeds. The strength of genetic divergence between breeds may vary dependent on the age of the breed, the scenario under which it emerged and the strength of reproductive isolation it has from other breeds. In this study, we investigated the Gulf Coast Native breed of sheep to determine if it contains lines of animals that are sufficiently divergent to be considered separate breeds. Allele sharing and principal component analysis (PCA) using nearly 50,000 SNP loci revealed a clear genetic division that corresponded with membership of either the Florida or Louisiana Native lines. Subsequent analysis aimed to determine if the strength of the divergence exceeded that found between recognised breed pairs. Genotypes from 14 breeds sampled from Europe and Asia were used to obtain estimates of pair-wise population divergence measured as *F*_ST_. The divergence separating the Florida and Louisiana Native (*F*_ST_ = 6.2%) was approximately 50% higher than the average divergence separating breeds developed within the same region of Europe (*F*_ST_ = 4.2%). This strongly indicated that the two Gulf Coast Native lines are sufficiently different to be considered separate breeds. PCA using small SNP sets successfully distinguished between the Florida and Louisiana Native animals, suggesting that allele frequency differences have accumulated across the genome. This is consistent with a population history involving geographic separation and genetic drift. Suggestive evidence was detected for divergence at the *poll* locus on sheep chromosome 10; however drift at neutral markers has been the largest contributor to the genetic separation observed. These results document the emergence of populations that can be considered separate breeds, with practical consequences for bio-conservation priorities, animal registration and the establishment of separate breed societies.

## Introduction

Sheep were first domesticated around 11,000 years ago for access to meat, before specialised breeds were subsequently developed to match animals to their captive environments. Today there are more than 1400 sheep breeds [1] containing considerable diversity in morphology, productive performance, size, shape and colour. The evolutionary history of breed development is of interest, and Menotti-Raymond and colleagues [2] describe four scenarios that may give rise to animal breeds. Firstly, ‘natural’ breeds are populations which became geographically dispersed following domestication and have subsequently undergone genetic drift and adaptation to their local environment. These are old breeds and often remain unmanaged, with the Soay and Gute breeds of sheep as good examples. Secondly, ‘established’ breeds are those that have undergone a managed process of human mediated selection towards a breed standard. Most economically relevant sheep breeds, such as the Merino, are considered to be established. A third scenario may give rise to ‘mutation’ breeds and involves the propagation of a specific desirable phenotype that distinguishes animals from their ancestors. This is most commonly focussed on pigmentation traits (e.g. Red Engadine, Swiss Black-Brown Mountain sheep) or horn type (e.g. Poll Dorset that have no horns and Jacob sheep which have either 4 or even 6 horns). The fourth scenario involves ‘hybrid’ breeds where deliberate crosses have been engineered between established breeds. The dual-purpose Perendale is an example that arose through the inter-breeding of Cheviot and Romney in New Zealand. Given this range of processes it is reasonable to anticipate the degree of genetic separation that distinguishes breeds will be highly variable. This has been demonstrated by previous investigations into the divergence between sheep breeds based on microsatellites [3], [4], SNP [5] and the mitochondrial genome [6]–[8].

Knowledge describing the strength of genetic division that exists between breeds has a number of important applications. Firstly, the degree of divergence can be used to direct prioritisation of resources available for the conservation of biodiversity [9], [10]. For example, where a high level of genetic differentiation is identified separating a phenotypically similar pair of breeds, high priority for conservation of animal genetic resources may be given to each. Conversely, phenotypically distinct ‘mutation’ breeds found to be genetically indistinguishable are less likely to individually attract a high priority for conservation. Beyond biodiversity, the relationship between breeds has practical implications for the delivery of emerging approaches to achieve genetic gain in livestock. Genomic selection is currently being implemented to speed genetic gain through the forward prediction of phenotypic performance on the basis of genotypic data alone [11]. The success of across breed genomic prediction will, in part, be determined by the relatedness between breed pairs. It is also important to recognise that genetic division may exist within a breed. Population substructure, when undetected, has the potential to generate spurious associations in experiments seeking to identify disease or production genes [12], [13]. The successful identification of population stratification, however, can be used to minimise inbreeding in closed populations [14].

Where substantial genetic separation has been identified between subpopulations within a breed, it becomes relevant to investigate if the divergence is sufficient to consider the subpopulations as separate breeds. This may trigger the establishment of separate breed societies to manage animal recording and accommodate divergent breeding objectives. It is important to note that the categorization of animals into breeds has a lot to do with non-genetic factors as diverse as human cultural identity, history and politics. This might explain why surprisingly little has been published concerning the minimum divergence required to declare subpopulations as separate breeds, given genetic distinction is not the sole determinant. While recognising non-genetic factors are important, the recent International Sheep Genomics Consortium's (ISGC) HapMap and Breed Diversity experiment offers the opportunity to explore this question at previously unattainable resolution through the use of 49,034 SNP [15]. We investigated the Gulf Coast Native (GCN), a breed adapted to the heat and semi tropical environment of the south-eastern part of the United States. The exact genetic origin of Gulf Coast sheep is unknown, however the Gulf Coast Native breed is considered to have descended from the Spanish Churra which were commonly brought to the Americas as early as the 1500s. The wool characteristics of modern Gulf Coast sheep indicate a contribution by Merino and/or British white faced breeds. Since its introduction the Gulf Coast Native breed has adapted to an environment characterised by high parasite loads, and a number of studies have investigated the genetic basis of their natural resistance to intestinal nematodes [16]–[19]. The majority of Gulf Coast animals lack wool on their faces, legs and bellies and most rams and some ewes carry horns, although box sexes may be polled. Size varies with rams weighing between 125–200 pounds and ewes 90–160 pounds. Importantly for this study, the breed contains two lines known as the Florida Native and the Louisiana Native. Anecdotal information suggests the two lines may have been founded by separate importations of animals and that phenotypic differences exist. The Louisiana Native are found predominantly in Arkansas, Louisiana, Missouri, Mississippi and Texas and may have arrived with explorers from Latin America. They are almost totally white with only limited pigmentation. The Florida Native are found predominantly in Alabama, Florida and Georgia and likely derived from sheep arriving with settlers to the east coast of Florida. They are mostly polled and white, but black and brown body coloring is common on their face and legs (Miller, pers comm). The Louisiana Native tend to have shorter legs and a larger body than the Florida Native. Resource flocks were maintained at Louisiana State University and the University of Florida for several years, and the census size for each line numbers only in the thousands, across less than 50 flocks each.

In this study, we first evaluated the ability of a dense set of SNP markers to detect population substructure within the Gulf Coast Native breed while ignoring line membership. Further, we calibrated the strength of substructure detected against a series of population comparisons to evaluate if the lines could be considered sufficiently diverse to be declared separate breeds. In the process the results document the emergence of domestic breeds and provide insights into the mechanisms involved.

## Results

### Strong Genetic Substructure Detected within the Gulf Coast Native

**Figure 1:**
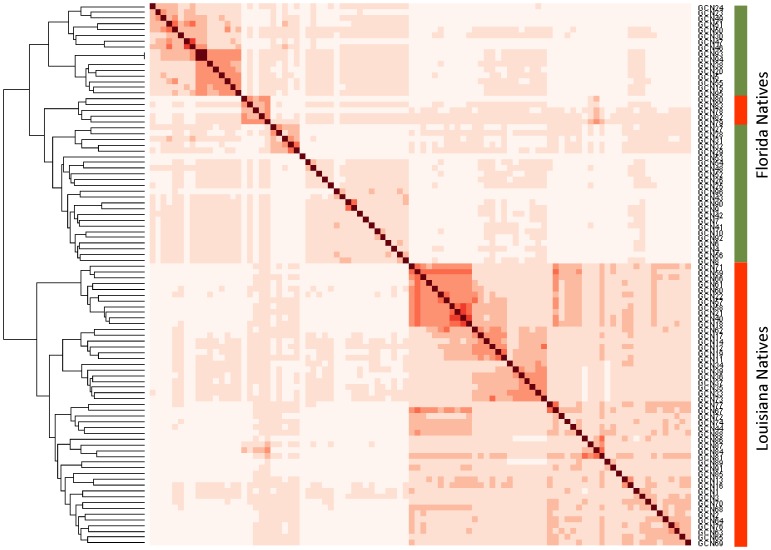
Allele sharing heat map of the Gulf Coast Native. The proportion of alleles shared (*A*_s_) between individuals was used to construct an ordered heat-map and dendogram. Cell colour represents the strength of allele sharing, where darker color indicates increasing allele sharing and relatedness between animals. As a guide, the lightest colors represent *A*_s_ values <0.65 and the darkest colors *A*_s_ values >0.80. Samples compared against themselves appear on the diagonal with the maximum *A*_s_ value of 1 and darkest colour. Two blocks were revealed that correspond to membership of either the Florida Native (indicated at right in green) or Louisiana Native (red).

**Figure 2:**
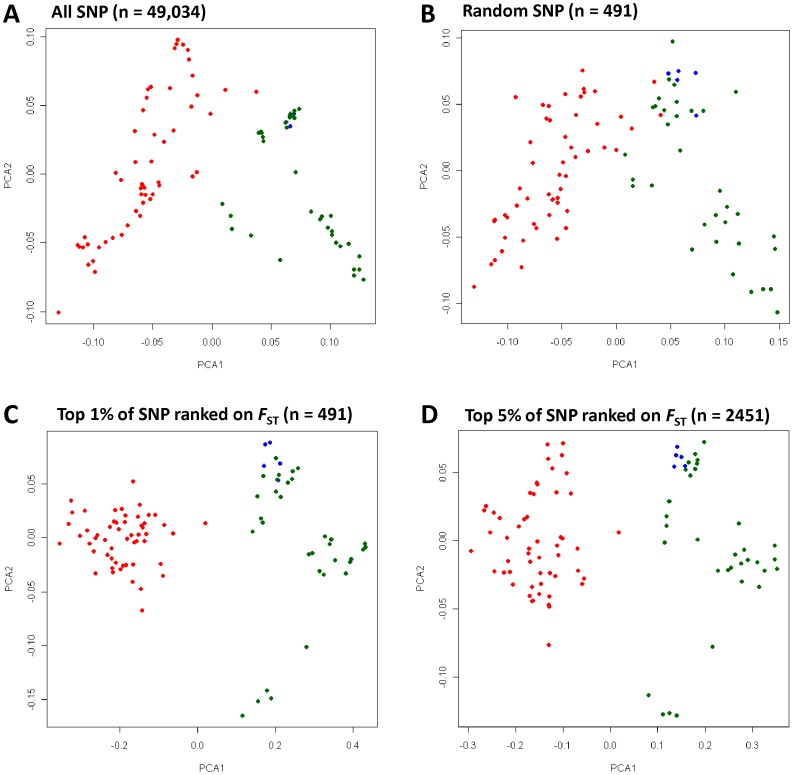
Clustering of animals based on principal component analysis of allele sharing. Individual animals were plotted for the first (PCA1) and second (PCA2) principal components using colours to distinguish the Louisiana Natives (red), Florida Natives (green) and Cracker (blue) lines. Four SNP sets were used to explore the effect of marker number and divergence on clustering: (**A**) all 49,034 SNP; (**B**) a random sample of 491 (1%) of SNP; (**C**) the top 1% of SNP ranked using *F*_ST_; (**D**) the top 5% of SNP ranked using *F*_ST_. Note that the scale for PCA1 differs between panels and that a larger proportion of the variation is captured by PCA1 using *F*_ST_ ranked SNP (C) compared with a random selection of markers (B).

To examine the relationship between Gulf Coast Native animals and search for evidence of population substructure, the average proportion of alleles shared between individuals was calculated using 49,034 SNP. The resulting 2×2 allele sharing (*A*_S_) matrix, when visualised as an ordered heat-map, revealed two distinct groups of animals within the breed (Figure 1). Animals within each group were, on average, more related to each other compared to a member of the second group. Inspection of the animals contained within each group revealed almost complete correspondence with membership of two established lines, the Florida Native and the Louisiana Native. The majority of Louisiana Natives formed a block displaying elevated allele sharing (lower right quadrant, Figure 1) that was separate from the Florida Natives (upper left quadrant). The top right quadrant of Figure 1 shows allele sharing between animals from different lines. This indicated five Louisiana Natives that grouped within the Florida Native block retain elevated allele sharing with other Florida Natives. To further explore the relationship between animals, principal components analysis (PCA) of allele sharing was performed to examine the clustering pattern within the population. Based on the full set of 49,034 SNP, animals from the two breed lines took non-overlapping positions in a plot of the two largest principal components (PCA1 and PCA2, Figure 2A). The Louisiana Natives had generally negative PCA1 values, while the Florida Natives had positive values. To test if the divergence detected between lines was the result of using a large number of markers, the analysis was repeated using a randomly selected set of 491 SNP (or 1% of the total). Individuals from the two lines again formed distinct clusters; however, the division between the groups was not complete when using a limited set of SNP (Figure 2B). The five Louisiana Natives that appeared among the Florida Native in the ordered heat-map (Figure 1) took at intermediate position in the PCA plot where animals from the two lines were indistinguishable.

### Are the Lines Sufficiently Divergent to be Considered Separate Breeds?

The degree of divergence observed between the Florida and Louisiana Natives was compared against the divergence that exists between populations recognised as separate breeds. The metric used was population *F*_ST_, which provides a single value for the degree of differentiation by averaging across SNP. A collection of 12 populations with SNP50 genotypes were drawn from the International Sheep Genomics Consortium (ISGC) HapMap and Breed Diversity experiment [15]. The genetic diversity within each breed, measured as heterozygosity (*H*_e_) and the proportion of polymorphic SNP (*P*_n_), is given in Table S1. The 12 populations were used to calculate *F*_ST_ at four levels: a) selection lines within the same breed (e.g. Meat and Milk Lacaune); b) separate breeds developed in the same region of Europe (e.g. Merino and Rambouillet); c) separate breeds developed in different regions of Europe (e.g. Merino and Poll Dorset) and d) separate breeds developed in either Europe or Asia (e.g. Merino and Tibetan). SNP genotypes from a total of 921 animals were used in a single analysis to estimate the divergence between every combination of the populations. The breeds used, the number of animals and the *F*_ST_ separating each population pair are listed in Table 1. As expected, divergence increased for each of the four categories. Selection lines within breed had the lowest average population divergence (1.7% from 2 comparisons, Table 2). Divergence increased to 4.2% for closely related breeds that were developed in southern Europe (15 comparisons, Table 2). Average divergence was higher again between breeds from Southern and Northern Europe (11.4%) and highest between breeds developed in Europe and Asia (13.6%, Table 2). These values provided calibration points for the interpretation of the genetic division between the Florida and Louisiana Native populations which had population *F*_ST_, = 6.2% (Tables 1 and 2). This is approximately three times higher than for selection lines within other breeds, and approximately 50% higher than the average divergence for breeds developed in Southern Europe.

**Table 1:** Population divergence measured as *F*_ST_ (%).

				Pair-wise Population Divergence Measured as F_ST_ (SD)^2^													
Population	Code	Origin^1^	Animals	FLN	LUN	MER	APD	APM	CAS	CHU	MEL	MIL	OJA	RAM	RAS	SUM	TIB
Florida Native	FLN	SE	40	0	6.2	5.4	10.3	5.3	5.2	6.5	5.4	6.1	4.8	6.9	4.0	14.5	11.0
Louisiana Native	LUN	SE	54	0.8	0	7.5	12.4	7.4	7.3	8.8	7.5	8.3	7.0	9.0	6.1	16.9	13.2
Merino	MER	SE	88	0.7	1.0	0	11.4	1.0	4.0	5.4	4.5	5.2	3.7	5.3	2.7	14.4	11.1
Poll Dorset	APD	NE	108	1.3	1.6	1.5	0	11.2	11.1	12.4	11.1	12.0	10.7	13.0	10.0	20.6	16.9
Poll Merino	APM	SE	98	0.7	1.0	0.3	1.5	0	3.9	5.3	4.4	5.1	3.6	5.0	2.6	14.1	10.9
Castellana	CAS	SE	23	0.7	0.9	0.5	1.4	0.6	0	4.5	4.0	4.7	2.9	5.9	2.1	13.7	10.4
Churra	CHU	SE	120	1.0	1.1	0.7	1.6	0.8	0.7	0	5.3	6.0	4.0	7.2	3.5	15.3	12.0
Meat Lacaune	MEL	CE	78	0.7	0.9	0.6	1.4	0.6	0.6	0.8	0	2.3	3.7	6.4	2.7	14.5	11.2
Milk Lacaune	MIL	CE	103	0.7	1.0	0.6	1.5	0.7	0.6	0.9	0.3	0	4.4	7.1	3.3	15.2	11.8
Ojalada	OJA	SE	24	0.7	1.0	0.6	1.4	0.6	0.5	0.6	0.6	0.6	0	5.5	1.8	13.6	10.3
Rambouillet	RAM	SE	102	1.0	1.3	0.9	1.7	0.7	1.1	1.1	1.0	1.1	1.0	0	4.4	16.4	13.2
Rasa Aragonesa	RAS	SE	22	0.6	0.9	0.5	1.4	0.5	0.4	0.6	0.4	0.5	0.4	0.9	0	12.7	9.5
Sumatra	SUM	A	24	1.6	1.7	1.6	2.1	1.6	1.6	1.7	1.6	1.6	1.6	1.8	1.5	0	13.2
Tibetan	TIB	A	37	1.2	1.4	1.3	1.9	1.3	1.3	1.4	1.3	1.3	1.3	1.6	1.2	1.6	0

1 The geographic region of breed development is given as SE (Southern Europe), NE (Northern Europe), (CE) Continental Europe or A (Asia).

2 *F*_ST_ (given as a percentage) is given above the diagonal and its standard deviation (SD) for each combination is given below (x1000).

**Table 2:** Average *F*_ST_ for different population comparisons.

Comparison	Populations Used	Number of Comparisons	Average_*F*_ST_ 1
Selection lines within breed	MER,APM,MEL,MIL	2	1.7
Breed pairs of Mediterranean origin	MER,CAS,CHU,OJA,RAM,RAS	15	4.2
Breed pairs of Southern vs Northern European origin	APD,MER,CAS,CHU,OJA,RAM,RAS	6	11.4
Breed Pairs of Asian vs European Origin	SUM,TIB,APD,MER,CAS,CHU,OJA,RAM,RAS	14	13.6
Florida Native v Louisiana Native	FLN,LUN	1	6.2

1 Average *F*_ST_ (percent) was calculated across comparisons.

### The Genetic Basis of Differentiation Separating Gulf Coast Native Lines

**Figure 3:**
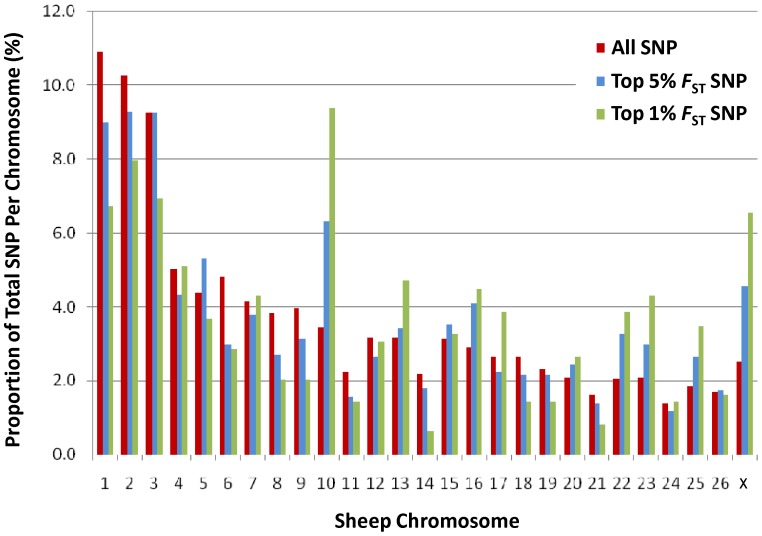
Distribution of divergent SNP across the sheep chromosome. The degree of divergence between the Florida and Louisiana Native populations was estimated for each SNP as *F*_ST_. The genomic distribution of SNP with extreme values (either the top 1% or 5% of ranked values) is shown as a function of their chromosomal location. This revealed an over representation of high *F*_ST_ SNP on chromosome 10 when compared to the proportion expected.

Analysis was performed to identify the subset of SNP which contributed most to the observed divergence between the Florida and Louisiana Native. SNP were ranked using *F*_ST_ to identify the top 1% (491 SNP) and top 5% (2451 SNP) of markers. To ensure ranking on *F*_ST_ did indeed enrich for SNP explaining the divergence between lines, individuals were clustered using the top 5% and top 1% of *F*_ST_ ranked SNP (Figure 2C and D). This check confirmed that *F*_ST_ ranked SNP clearly delineated the lines much more strongly than a marker panel of the same size selected at random (Figure 2B and 2C). Plotting the genomic distribution of SNP with extreme *F*_ST_ revealed two chromosomes (OAR10 and OARX) contained an excess compared to the number expected based on chromosome size (Figure 3). Chromosome 10 contained 9.2% of the top 1% *F*_ST_ SNP which is more than double the proportion expected (3.8%). Chromosome 10 is of particular interest given it harbours the *Poll* locus responsible for the absence of horns [15], [20] and matches to the anecdotal evidence suggesting a higher prevalence of polled animals within Florida Native compared with Louisiana Native (Miller, pers com). This finding prompted analysis of the *Poll* locus to compare haplotype frequencies present within the Australian Poll Merino, Merino, Florida Native and Louisiana Native. Genotypes at four SNP spanning 77 Kb at the *Poll* locus were phased into haplotypes, and their frequency is given in Table 3. Haplotype H1, known to be associated with selection for polled animals [15], was present at high frequency in both the Australia Poll Merino (0.57) and Florida Native (0.49). In contrast, haplotype H1 was at low frequency in the Merino (0.09) and Louisiana Native (0.19, Table 1), suggesting selection for the absence of horns has contributed to the divergence between the Florida and Louisiana Native populations.

**Table 3:** Haplotype frequencies at the *Poll* locus on sheep chromosome 10.

Population	Animals	Haplotypes^1^	Haplotype Frequency^2^			
			H1	H2	H3	Other
Poll Merino	98	6	0.57	0.24	0.12	0.07
Merino	88	5	0.09	0.58	0.13	0.20
Florida Native	35	6	0.49	0.03	0.39	0.09
Louisiana Native	54	3	0.19	0.00	0.57	0.24

1 Haplotypes were constructed using SNP genotypes at four loci (*OAR10_29469450*, *OAR10_29511510*, *OAR10_29538398* and *OAR10_29546872*) that span 77 Kb at the *Poll* locus on sheep chromosome 10. The total number of haplotypes observed is given for each population.

2 The most frequently observed haplotypes were labelled H1–H3. Haplotype 1 (H1) comprised alleles GGAA at the four SNP ^1^ and was associated with selection for the absence of horns in the ISGC HapMap study [15]. Haplotypes H2 and H3 consisted of alleles AGGT and GGAT respectively. The combined frequency of all other haplotypes observed within each population is given as ‘other’.

### Genetic Relationship Linking the Gulf Coast Native to Other Breeds

**Figure 4:**
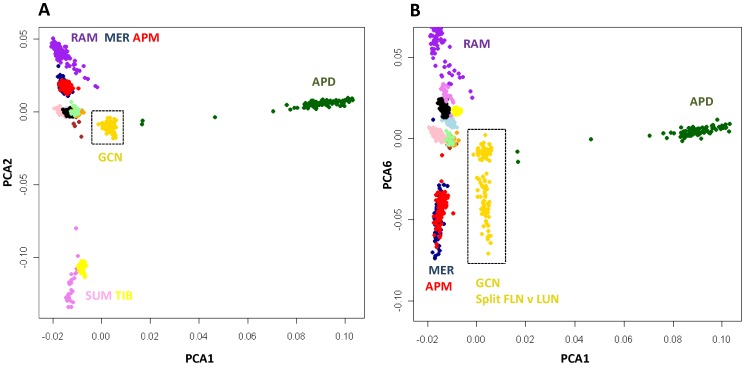
Genetic relationship between Gulf Coast Native and 12 other breeds. Individuals were clustered using PCA of allele sharing and plotted for PCA1 and 2 (**A**) and PCA1 and 6 (**B**). Individuals from different breeds are given using different colored symbols as follows: Gulf Coast Native (GCN gold), Merino (MER dark blue), Poll Dorset (APD dark green), Poll Merino (APM red), Castellana (CAS brown), Churra (CHU pink), Meat Lacaune (MEL light blue), Milk Lacaune (MIL black), Ojalada (OJA orange), Rambouillet (RAM purple), Rasa Aragonesa (RAS pale green), Sumatra (SUN violet) and Tibetan (TIB yellow). The two largest principal components (**A**) separated Asian (SUM, TIB) and Northern European animals (APD) from a cluster containing breeds developed in southern Europe (including RAM, MER and APM). Plotting PCA1 and PCA6 revealed the genetic division within GCN (**B**).

The population history of the Gulf Coast Native was explored by comparing them to a selection of four Spanish breeds, a meat type breed from the United Kingdom and two Asian breeds that served as outgroups. Allele sharing was calculated between 921 animals (Table S1) and used to construct a PCA plot (Figure 4). Gulf Coast Native individuals were positioned separately from all other breeds (Figure 4) in a cluster near a group of Spanish breeds including the Rasa Aragonesa, Ojalada and Castellana. The GCN grouped away from the Poll Dorset (Northern European) and Asian derived animals.

## Discussion

Breeds are human constructs that assist in producing animals with a uniform phenotype and populations that have desirable attributes. Over the last few hundred years, the emergence of new breeds has most often occurred under the ‘mutation’ or ‘hybrid’ scenario where man intentionally develops new breeds based on phenotype [2]. Under these scenarios, the establishment of a new breed is not based on prior knowledge concerning genetic division or divergence within a population, although this is the long term consequence of erecting reproductive barriers within a population. The two remaining scenarios for breed development are emergence of ‘natural’ or ‘established’ breeds. If these events have occurred in the distant past, the expectation is that they will be characterised by genetic separation and division. In this framework, we sought to explore a specific case where a breed has been maintained over several hundred years where multiple lines of animals exist, but where no declaration has been made concerning their genetic eligibility to be termed separate breeds. To search for evidence for genetic division, a set of nearly 50,000 SNP markers were genotyped in the Florida and Louisiana Native lines, and the divergence separating them expressed as *F*_ST_. To generate a type of calibration curve of *F*_ST_ values, other breeds were used that are expected to have increasing genetic separation. At one extreme pairs were examined using lines within the same breed while at the other extreme pairs were examined using breeds developed in Europe and Asia. These comparisons provided a guide to the percent values for *F*_ST_ to be expected when calculated using 50,000 SNP, and the values increased in a step-wise fashion for the four comparison types (Table 2). Importantly, the divergence observed between Florida and Louisiana Natives (*F*_ST_ = 6.2%) was higher than the average value separating recognised breed pairs developed in Mediterranean Europe (average *F*_ST_ = 4.2%, Table 2). The Australian Merino and American Rambouillet are an example that contributed to the average value of Mediterranean derived breeds. Both were originally developed in southern Europe before being exported and subsequently adapted to production systems in Australia and the US, respectively. They have pair-wise population *F*_ST_ of 5.3% which is lower than for the Florida and Louisiana Native (Table 2). Our interpretation, therefore, is that on the basis of genetic data the two lines of Gulf Coast sheep can be considered as different breeds.

Analysis of the divergence between subpopulations suggests the emergence of separate breeds has occurred in a manner that most closely resembles the ‘natural’ scenario [2]. Specifically, there has been some degree of geographic separation (Florida versus Louisiana) followed by genetic drift within each separated population. This conclusion is based on the observation that PCA was able to reconstitute the genetic division between the Gulf Coast populations when performed using a random subset containing 1% of available SNP. This indicates allele frequency differences are present at the majority of SNP, rather than at a small number of markers in response to human mediated selection pressure. Suggestive evidence was detected for selection at the horn/poll locus; however, the difference in haplotype frequency was modest and did not contribute a meaningful amount to the total divergence observed. Furthermore, the divergence does not appear to have involved a strong founder effect whereby a small number of animals were used to create the two lines. Founder effects are accompanied by restricted genetic diversity; however, the proportion of SNP displaying polymorphism and expected heterozygosity observed within Gulf Coast animals was amongst the highest of any population tested (Table S1). In summary, the emergence of these new breeds is most consistent with the natural scenario, as opposed to the foundation of new breeds by either the ‘mutation’ or ‘hybrid’ scenarios.

## Materials and Methods

### Animal Material

Gulf Coast Natives were sampled from a total of eleven breeders distributed across six states in the south-eastern region of the United States. Florida Natives (n = 35) samples were from six breeders distributed across Florida, Georgia and Texas, while the Louisiana Natives samples were collected from six breeders in Arkansas, Louisiana, Missouri and Texas. Five animals were sampled from a Florida flock of the ‘cracker’ line, and these are annotated separately from the Florida Native and Louisiana native individuals in Figure 2. Given sampling was performed across a number of different American States and within different flocks, the animals used can be considered representative of the two lines. Owners of all animals used in the study gave permission for the collection of blood prior to sampling. Animal handling procedures including the collection of blood samples was submitted to the Louisiana Statue University Agricultural Center Institutional Animal Care and Use Committee (IACUC) and approved on June 16^th^ 2005 under Protocol number AE AE 05-12. Details for all of the other breeds used have been described elsewhere [15].

### Genotyping and Quality Control

All DNA samples were genotyped using the Illumina Ovine SNP50 BeadChip as part of the ISGC HapMap and Breed Diversity experiment [15]. SNP genotype calls were evaluated by a series of quality control filters to remove poor performing samples and 5207 SNP that failed to meet any of five quality criteria [15]. A total of 49,034 SNP remained and were used in this study.

### Genetic Diversity and Analysis of Population Substructure

Within breed diversity was calculated as the proportion of polymorphic SNP (*P*_n_) and gene diversity (*H*_e_) from the full SNP dataset using PLINK v2.05 [21]. Allelic richness (*A*_r_), which measures the normalised number of alleles and private allelic richness (p*A*_r_), which gives a measure of population distinctiveness, were calculated using ADZE [22]. The average proportion of alleles shared between animals (*A*_s_) was calculated as (IBS2+0.5*IBS1/N) where IBS1 and 2 are the number of loci that share either 1 or 2 alleles and N is the number of SNP pairs tested. These values were calculated using PLINK v2.05 [21] through use of commands - - cluster and - - distance-matrix. The resulting matrix of *A*_s_ values was used to generate an ordered heatmap and dendogram using R software package RColorBrewer. Principal components analysis (PCA) of *A*_s_ values was performed using smartpca implemented in Eigensoft [23]. Population divergence was calculated for each SNP as *F*_ST_ and as global *F*_ST_ using the methods of Nicholson and colleagues [24].

### Analysis of the Poll Locus

SNP50 genotypes from four populations were used for the analysis of the *Poll* locus; the Florida Native, Louisiana Native, Poll Merino and Merino. The Poll Merino (98 animals with no horns) and Merino populations (88 animals with horns) were collected as unrelated industry sires in Australia as part of the ISGC HapMap experiment [15]. Haplotypes were constructed and their population frequencies estimated using Haploview [25] for four SNP (*OAR10_29469450*, *OAR10_29511510*, *OAR10_29538398* and *OAR10_29546872*) which span the *Poll* locus on sheep chromosome 10 [15], [18].

## Supporting Information

Table S1 **Basic indices of genetic diversity measured within breed.** Breeds are listed with decreasing expected heterozygosity or gene diversity (*H*_e_). Other measures include the proportion of SNP displaying polymorphism (*P*_n_); the inbreeding coefficient (F); allelic richness (*A*_r_) and private allele richness (p*A*_r_). These results are taken from the ISGC HapMap and Breed Diversity Experiment [15].
(DOC)
