# Supplementary material for: Tracking the Emergence of a New Breed Using 49,034 SNP in Sheep
Source: PLoS One. 2012 Jul 27;7(7):e41508. doi: 10.1371/journal.pone.0041508 (PMC3407242; doi:10.1371/journal.pone.0041508)
Supplement: Table S1 — Basic indices of genetic diversity measured within breed. Breeds are listed with decreasing expected heterozygosity or gene diversity (H e). Other measures include the proportion of SNP displaying polymorphism (P n); the inbreeding coefficient (F); allelic richness (A r) and private allele richness (pA r). These results are taken from the ISGC HapMap and Breed Diversity Experiment [15]. (DOC) [file pone.0041508.s001.doc]

**Supplementary Table 1**

Basic indices of genetic diversity measured within breed.

|  |  |  |  |  |  |
| --- | --- | --- | --- | --- | --- |
|  |  |  |  |  |  |
| Breed | *H*_e_ | *P*_n_ | F | *A*_r_ | p*A*_r_ |
|  |  |  |  |  |  |
|  |  |  |  |  |  |
| Gulf Coast Native | 0.38 | 0.96 | 0.09 | 1.99 | 0.01 |
| Rasa Aragonesa | 0.38 | 0.95 | 0.04 | 1.98 | 0.01 |
| Australian Industry Merino | 0.37 | 0.96 | 0.10 | 1.99 | 0.00 |
| Australian Poll Merino | 0.37 | 0.96 | 0.09 | 1.99 | 0.01 |
| Castellana | 0.37 | 0.94 | 0.07 | 1.97 | 0.01 |
| Meat Lacaune | 0.37 | 0.96 | 0.10 | 1.99 | 0.01 |
| Ojalada | 0.37 | 0.95 | 0.06 | 1.98 | 0.00 |
| Churra | 0.36 | 0.96 | 0.11 | 1.99 | 0.00 |
| Milk Lacaune | 0.36 | 0.96 | 0.11 | 1.99 | 0.00 |
| Rambouillet | 0.36 | 0.96 | 0.14 | 1.99 | 0.00 |
| Australian Poll Dorset | 0.34 | 0.95 | 0.15 | 1.98 | 0.01 |
| Tibetan | 0.34 | 0.92 | 0.20 | 1.95 | 0.04 |
| Sumatran | 0.31 | 0.89 | 0.24 | 1.91 | 0.04 |
|  |  |  |  |  |  |

Breeds are listed with decreasing expected heterozygosity or gene diversity (*H*_e_). Other measures include the proportion of SNP displaying polymorphism (*P*_n_); the inbreeding coefficient (F); allelic richness (*A*_r_) and private allele richness (p*A*_r_). These results are taken from the ISGC HapMap and Breed Diversity Experiment [15].
